# Pathological features and their prognostic impacts on oral cavity cancer patients among different subsites – A singe institute’s experience in Taiwan

**DOI:** 10.1038/s41598-017-08022-w

**Published:** 2017-08-07

**Authors:** Shih-An Liu, Chen-Chi Wang, Rong-San Jiang, Fang-Yi Lee, Wen-Jiun Lin, Jin-Ching Lin

**Affiliations:** 1 Department of Otolaryngology, Taichung Veterans General Hospital, Taichung, 40705 Taiwan; 2 Department of Pathology, Taichung Veterans General Hospital, Taichung, 40705 Taiwan; 3 Department of Radiation Oncology, Taichung Veterans General Hospital, Taichung, 40705 Taiwan; 4 Faculty of Medicine, School of Medicine, National Yang-Ming University, Taipei, 11221 Taiwan; 5 Department of Medical Research, China Medical University Hospital, China Medical University, Taichung, 43302 Taiwan

## Abstract

We investigated the relationship of different primary subsites together with their pathological features on the survival of oral cavity squamous cell carcinoma (OCSCC) patients. We retrospectively reviewed OCSCC patients and documented their demographic data, pathological features and clinical outcome. The Cox proportional hazard model was used to examine the influence of various pathological features on the prognosis in different subsites of oral cavity. There were totally 1,383 OCSCC patients enrolled for final analysis. Perineural invasion had a poor prognosis at the early stage of OCSCC patients especially those with primary at the tongue. In addition, lymphovascular invasion was associated with poor survival at the late stage especially those with primary at the buccal mucosa and the tongue. The impact of pathological features on the survival of OCSCC patients varied in different subsites. Further investigation is warranted to validate our finding in a multicenter study. Grouping the different markers to establish a prognostic scoring system may provide more accurate evaluation of the prognosis in OCSCC patients.

## Introduction

Squamous cell carcinoma (SCC) is the most common histological type in oral cavity cancer. In fact, it is almost synonymous with oral cavity cancer as it forms nearly 95% of all malignant lesions found in the oral cavity^1^. In 2012, approximately 300,000 new cases were diagnosed worldwide with 145,400 mortalities^2^. No marked progress in the management of oral cavity cancer has been made in recent years. Although the combinations of therapeutic protocols has improved the patient’s quality of life, the 5-year survival rate remains unchanged^3, 4^.

The most important and well-known prognostic factor of oral cavity squamous cell carcinoma (OCSCC) is the tumor-node-metastasis (TNM) system^5^. The worse prognosis is for patients presented with the late TNM stage. In terms of its pathological characteristics, histological grade is known to be associated with distant metastasis^6^. A previous study revealed that early tongue cancer with an invasion depth >=4 mm had a high loco-regional recurrent rate and a poor prognosis^7^. The prognostic value of perineural invasion (PNI) is also documented^8^. PNI is associated with neck recurrence and poor disease-specific survival (DSS) in patients with T1 oral SCC^9^. In particular, lymphovascular invasion (LVI) has increased death rate^10^. Furthermore, extracapsular spread (ECS) doubles the incidence of local recurrence and distant metastasis^11^. Finally, patients with PNI, LVI, and ECS simultaneously have the worst prognosis^12^. Previous studies on oral cancer indicated that the prognostic factors and the failure patterns vary across different primary subsites^13, 14^. Despite of these findings, no report is yet available on the importance of pathological features at different primary subsites. Therefore, the purpose of this study was to investigate the importance of tumor grading, PNI, LVI, and ECS with reference to different primary subsites on the prognosis of OCSCC via multivariate analyses.

## Results

Comprehensive information was obtained from 1,383 patients. Their average age at diagnosis was 52.9 ± 11.1 years with 93.8% (n = 1,297) being male. The commonest primary site was the buccal mucosa (n = 556, 40.2%), followed by the tongue (n = 417, 30.2%). Pathological stage I disease was found in 378 patients (27.3%), whereas 204 (14.8%), 184 (13.3%), and 617 (44.6%) patients had pathological stage II, III, and IV diseases, respectively. In terms of pathological features, 122 patients (8.8%) had well differentiated (WD) SCC, whereas 932 patients (67.4%) and 329 patients (23.8%) had moderately differentiated (MD) and poorly differentiated (PD) SCC, respectively. PNI was identified in 314 patients (22.7%), while LVI and ECS were found in 360 patients (26.0%) and 230 patients (16.6%), respectively. The majority of patients had surgical margins more or equal to 5 mm (n = 1,148, 83.0%). There were 661 patients (47.8%) with adverse pathological features and adjuvant treatments were recommended. Among them, 654 patients (98.9%) actually underwent postoperative radiotherapy with/without chemotherapy. Death later occurred in 349 patients (25.2%) and 489 patients (35.4%) developed loco-regional recurrence. One hundred and thirty-three patients (9.6%) had distant metastasis. The average follow-up period was 42.8 (±28.3) months.

When patients were compared according to primary subsites, no significant difference was found in terms of their ECS, postoperative radiation, loco-regional recurrence, distant metastasis, and 5-year DSS. However, patients with primary at the floor of mouth (FOM) appeared to be younger and were mostly male. The highest proportions of PNI and LVI were found in patients with tongue cancer whereas the highest proportions of ECS and tumors with a surgical margin less than 5 mm were found in patients with FOM cancer. In summary, depending on the primary subsites involved, significant differences were found in terms of patients’ age, gender, personal habits, histological grade, PNI, LVI, surgical margin, and pathological stage. Details of these differences are shown in Table 1.

**Table 1:** Comparison of variables among subsites of oral squamous cell carcinoma patients.

	Lip (n = 54)	Gum (n = 218)	FOM (n = 33)	Tongue (n = 417)	Buccal (n = 556)	Palate (n = 74)	RMT (n = 27)	*P*
Age [Mean (SD)]	52.4(9.8)	55.9(11.6)	51.7(7.0)	51.3(11.3)	52.7(10.9)	55.2(11.8)	52.3(9.4)	<0.001
Gender [Male (%)]	53(98.1)	203(93.1)	37(100)	364(87.3)	541(97.3)	72(97.3)	27(100)	<0.001
Smoking (%)	48(88.9)	182(83.5)	35(94.6)	319(76.9)	504(90.6)	60(81.1)	26(96.3)	<0.001
Betel quid (%)	48(88.9)	177(81.2)	35(94.6)	289(69.3)	503(90.5)	58(78.4)	23(85.2)	<0.001
Alcohol (%)	39(72.2)	165(76.7)	36(97.3)	278(66.7)	413(74.3)	50(67.6)	20(74.1)	0.002
Histological grade								0.004
WD (%)	10(18.5)	16(7.3)	2(5.4)	22(5.3)	62(11.2)	9(12.2)	1(3.7)	
MD (%)	35(64.8)	161(73.9)	26(70.3)	280(67.1)	360(64.7)	47(63.5)	23(85.2)	
PD (%)	9(16.7)	41(18.8)	9(24.3)	115(27.6)	134(24.1)	18(24.3)	3(11.1)	
Perineural invasion (%)	11(20.4)	33(15.1)	9(24.3)	131(31.4)	119(21.4)	5(6.8)	6(22.2)	<0.001
Lymphvascular invasion (%)	7(13.0)	50(22.9)	12(32.4)	138(33.1)	132(23.1)	12(16.2)	9(33.3)	0.001
Extracapsular spread	8(14.8)	36(16.5)	11(29.7)	82(19.7)	79(14.2)	10(13.5)	4(14.8)	0.108
Surgical margin <5mm (%)	8(14.8)	43(19.7)	9(24.3)	45(10.8)	108(19.4)	18(24.3)	4(14.8)	0.004
Pathological stage								<0.001
Stage I and II (%)	35(64.8)	55(25.2)	14(37.8)	188(45.1)	235(32.3)	42(56.8)	13(48.1)	
Stage III and IV (%)	19(35.2)	163(74.8)	23(62.2)	229(54.9)	321(57.7)	32(43.2)	14(51.9)	
Postoperative radiation (%)	19(35.2)	95(43.6)	22(59.5)	210(50.4)	261(46.9)	33(44.6)	14(51.9)	0.196
Loco-regional recurrence (%)	16(29.6)	81(37.2)	16(43.2)	151(36.2)	187(33.6)	26(35.1)	12(44.4)	0.687
Distant metastasis (%)	3(5.6)	14(6.4)	5(13.5)	45(10.8)	59(10.6)	3(4.1)	4(14.8)	0.174
Cancer death (%)	11(20.4)	41(18.8)	10(27.0)	109(26.1)	151(27.2)	20(27.0)	7(25.9)	0.329
5-year DSS (all stage, %)	85.76	81.39	68.89	74.56	77.58	75.18	85.25	0.4530
Stage I and II (%)	88.91	87.19	90.91	86.23	86.41	77.78	100	0.8745
Stage III and IV (%)	54.14	72.66	43.10	53.70	55.78	64.28	51.43	0.0256

SD: standard deviation; WD: well differentiated; MD: moderately differentiated; PD: poorly differentiated; FOM: floor of mouth; RMT: retromolar trigone; DSS: disease-specific survival.

Univariate survival analysis based on subsites showed no difference in the age of diagnosis. Prognosis was poorer for those with MDSCC in the buccal mucosa compared to WDSCC and the hazard ratio (HR) was 4.47 (95% confidence interval [CI], 1.64–12.16). Similarly, poor prognosis was found with PDSCC in the buccal mucosa compared to WDSCC. The HR was 7.53 (95% CI, 2.71–20.87). PNI was associated with a low survival rate in patients with primary at the tongue (HR, 2.88; 95% CI, 1.97–4.20), the gum (HR, 2.84; 95% CI, 1.42–5.67), and the buccal mucosa (HR, 2.39; 95% CI, 1.70–3.36). LVI was related to a poor prognosis in all patients except those with primary at the FOM and RMT. Patients in the late stage tended to have a prognosis poorer than those in the early stage. Details are shown in Table 2.

**Table 2:** Univariate analysis of the impact of clinic-pathological features on the survival of oral squamous cell carcinoma patients based on subsites.

	Lip		Gum		Floor of mouth		Tongue		Buccal		Palate		RMT	
	HR	95% CI	HR	95% CI	HR	95% CI	HR	95% CI	HR	95% CI	HR	95% CI	HR	95% CI
Age >50 years	0.64	0.11–1.31	0.60	0.33–1.12	0.35	0.07–1.64	0.87	0.60–1.27	0.99	0.72–1.36	0.71	0.29–1.75	1.17	0.21–6.42
**Tumor grade**														
MD vs. WD	3.16	0.40–25.00	1.06	0.32–3.50	33.18	0–>10000	2.69	0.66–11.04	**4.47**	1.64–12.16	1.50	0.33–6.78	27.25	0–>10000
PD vs. WD	1.39	0.09–22.34	1.70	0.46–6.33	1.01	0–>10000	**7.35**	1.79–30.23	**7.53**	2.71–20.87	2.73	0.56–13.20	1.00	0–>10000
PNI	1.46	0.39–5.53	**2.84**	1.42–5.67	1.66	0.33–8.31	**2.88**	1.97–4.20	**2.39**	1.70–3.36	3.34	0.97–11.47	2.12	0.39–11.61
LVI	**5.05**	1.47–17.31	**4.81**	2.53–9.18	1.08	0.28–4.20	**4.13**	2.81–6.08	**3.23**	2.33–4.49	**3.62**	1.33–9.82	800.0	0.12–>10000
ECS	**3.97**	1.16–13.65	**2.33**	1.06–5.09	0.38	0.05–3.00	**4.16**	2.82–6.15	**3.03**	2.12–4.33	0.54	0.12–2.32	**7.77**	1.07–56.40
Margin <5 mm	3.56	0.94–13.54	**2.55**	1.30–5.02	**15.97**	3.01–84.79	**2.26**	1.39–3.68	**1.85**	1.28–2.69	0.95	0.35–2.62	0.04	0–7003
Late stage	**6.02**	1.59–22.80	**2.40**	1.01–5.73	**7.39**	0.94–58.33	**4.72**	2.95–7.57	**3.30**	2.25–4.85	**2.79**	1.11–7.02	85.89	0.11–67070

Abbreviations: RMT, retromolar trigone; HR, hazard ratio; CI, confidence interval; WD, well differentiated; MD, moderately differentiated; PD, poorly differentiated; PNI, perineural invasion; LVI, lymphovascular invasion; ECS, extra-capsular spread.

When adjusted for other variables, the survival impact was still found in histological grade on patients with primary at the buccal mucosa (PD vs. WD: HR, 3.70; 95% CI, 1.31–10.49). PNI was associated with a poor survival only in patients with primary at the tongue (HR, 1.54; 95% CI, 1.03–2.31). LVI was related to a poor prognosis in patients with primary at the gum (HR, 3.01; 95% CI, 1.31–6.91), the tongue (HR, 1.74; 95% CI, 1.08–2.81), and the buccal mucosa (HR, 1.86; 95% CI, 1.28–2.70). Primary with a margin less than 5 mm was related to a low survival rate only in patients with primary at the gum (HR, 2.95; 95% CI, 1.31–6.63) and the buccal mucosa (HR, 1.66; 95% CI, 1.12–2.46). Details data are listed in Table 3.

**Table 3:** Multivariate analysis of the impact of clinic-pathological features on the survival of oral squamous cell carcinoma patients based on subsites.

	Lip		Gum		Floor of mouth		Tongue		Buccal		Palate		RMT	
	HR	95% CI	HR	95% CI	HR	95% CI	HR	95% CI	HR	95% CI	HR	95% CI	HR	95% CI
Age >50 years	0.96	0.11–8.25	0.52	0.25–1.09	0	0–>10000	0.97	0.66–1.42	0.94	0.68–1.30	0.36	0.12–1.07	0.22	0–>10000
**Tumor grade**														
MD vs. WD	0.90	0.09–9.55	0.81	0.24–2.80	>1000	0–>10000	1.31	0.31–5.50	2.64	0.96–7.28	1.84	0.37–9.20	0.68	0–>10000
PD vs. WD	0.26	0.01–6.30	0.93	0.22–3.95	>1000	0–>10000	2.17	0.50–9.46	**3.70**	1.31–10.49	3.03	0.57–16.21	0.57	0–>10000
PNI	0.25	0.04–1.47	1.14	0.47–2.77	10.50	0.57–194.4	**1.54**	1.03–2.31	1.14	0.77–1.68	4.33	0.91–20.56	0.18	0.09–96.36
LVI	6.78	0.71–64.29	**3.01**	1.31–6.91	>1000	0–>10000	**1.74**	1.08–2.81	**1.86**	1.28–2.70	3.26	0.78–13.69	>1000	0–>10000
ECS	0.91	0.09–8.94	1.12	0.40–3.13	0	0–>10000	1.51	0.95–2.39	1.43	0.95–2.16	0.11	0.02–0.59	1.74	0.19–15.79
Margin <5 mm	4.45	0.44–44.88	**2.95**	1.31–6.63	3.34	0.53–21.08	1.45	0.88–2.40	**1.66**	1.12–2.46	1.10	0.34–3.53	0	0–>10000
Late stage	5.53	0.74–41.46	1.88	0.73–4.84	71.38	0.67–7580	**2.34**	1.35–4.05	**2.08**	1.36–3.18	**5.57**	1.75–17.74	1.99	0–>10000

Abbreviations: RMT, retromolar trigone; HR, hazard ratio; CI, confidence interval; WD, well differentiated; MD, moderately differentiated; PD, poorly differentiated; PNI, perineural invasion; LVI, lymphovascular invasion; ECS, extra-capsular spread.

**Figure 1:**
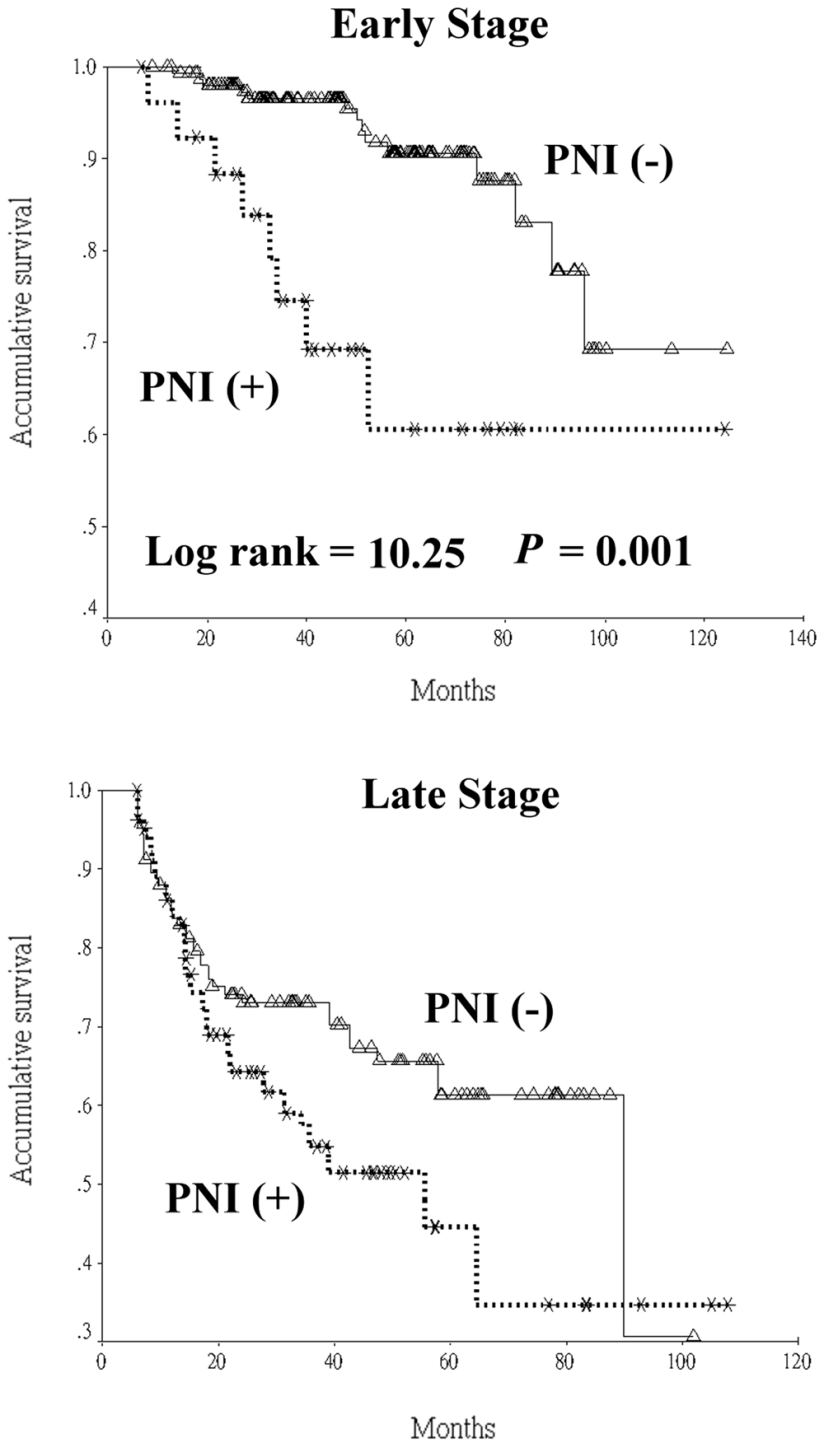
Univariate analysis of the presence of perineural invasion (PNI) on disease-specific survival stratified according to initial pathological stage in tongue squamous cell carcinoma patients.

**Figure 2:**
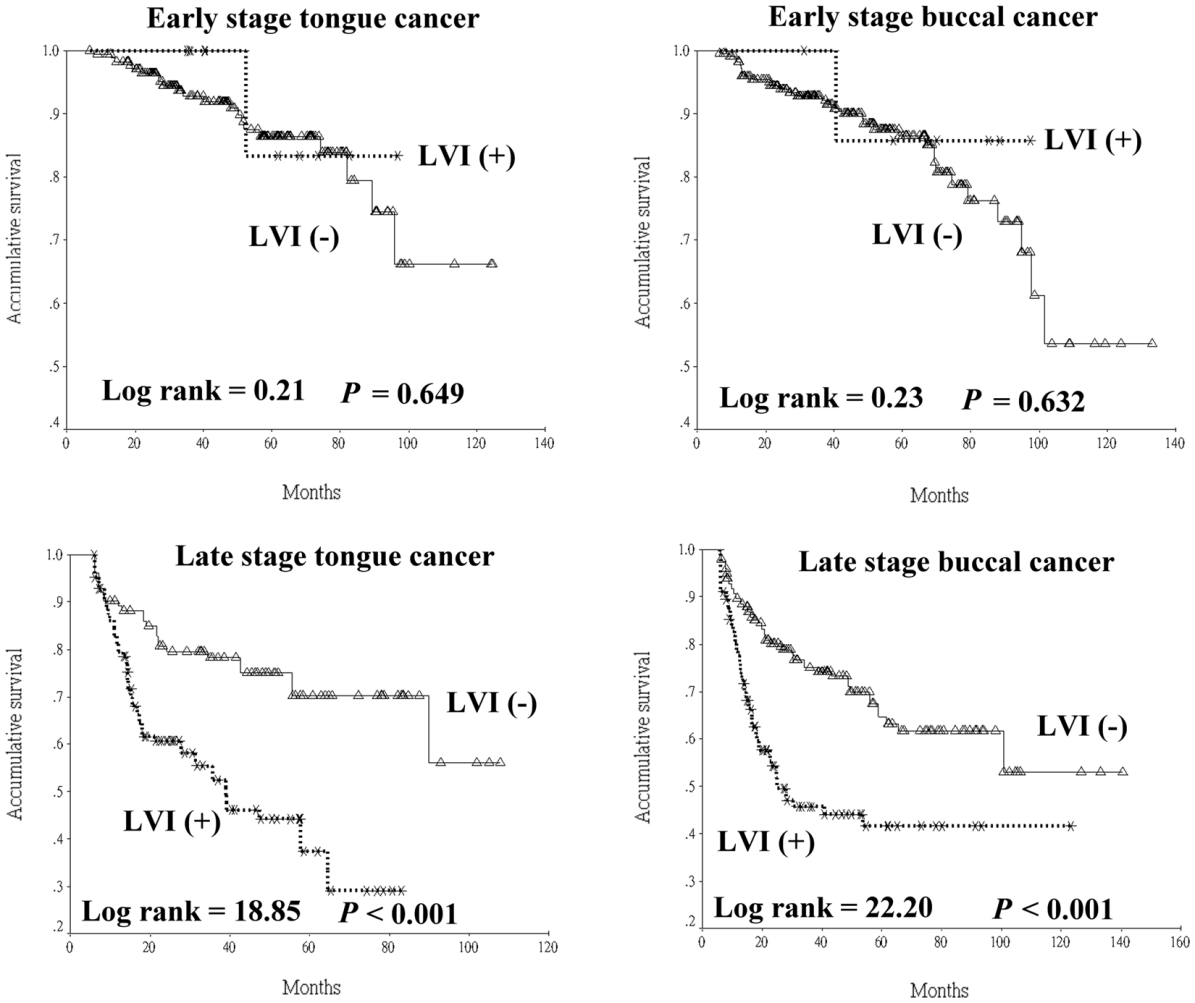
Univariate analysis of the presence of lymphovascular invasion (LVI) on disease-specific survival stratified according to initial pathological stage in tongue and buccal squamous cell carcinoma patients.

When data of all patients were analyzed with the Cox proportional hazard model, age and ECS were not associated with DSS. But histological grade, PNI, LVI, surgical margin and pathological stage were all independent factors related to DSS. When patients were divided based on the pathological stage, PNI was found to be independent prognosticator of DSS in stage I and II whereas LVI and surgical margin were independent prognosticators of DSS in stage III and IV. Limited by the number of patients, we were only able to analyze pathological features of those with primary sites at the tongue and the buccal mucosa. In tongue SCC, PNI was found to be the only significant factor associated with DSS of stage I and II (Fig. 1). Finally, in tongue SCC, LVI was associated with DSS of stage III and IV, and similarly with stage III and IV in buccal SCC (Fig. 2). Details are shown in Table 4.

**Table 4:** Multivariate analysis of the impact of clinic-pathological features on the survival of oral squamous cell carcinoma patients based on pathological stage and subsites (the tongue and the buccal mucosa only).

	All (n = 1383)		All Stage I & II (n = 582)		All Stage III & IV (n = 801)		Tongue Stage I & II (n = 188)		Tongue Stage III and IV (n = 229)		Buccal Stage I & II (n = 235)		Buccal Stage III & IV (n = 321)	
	HR	95% CI	HR	95% CI	HR	95% CI	HR	95% CI	HR	95% CI	HR	95% CI	HR	95% CI
Age >50 years	0.83	0.67–1.03	0.81	0.51–1.29	0.81	0.64–1.04	0.69	0.29–1.63	0.96	0.62–1.47	0.68	0.34–1.34	0.98	0.68–1.43
**Tumor grade**														
MD vs. WD	1.72	0.96–3.11	1.63	0.70–3.84	1.66	0.73–3.77	0.78	0.16–3.82	>1000	0–>10000	3.08	0.72–13.13	2.17	0.52–9.00
PD vs. WD	**2.30**	1.25–4.25	2.17	0.83–5.67	2.16	0.93–5.01	1.85	0.34–9.97	>1000	0–>10000	4.75	0.98–22.92	3.01	0.71–12.70
PNI	**1.36**	1.07–1.72	**2.36**	1.31–4.21	1.24	0.96–1.60	**3.81**	1.38–10.54	1.29	0.83–2.00	0.76	0.22–2.59	1.10	0.72–1.67
LVI	**2.05**	1.59–2.63	0.84	0.30–2.36	**2.13**	1.63–2.80	0.23	0.03–1.90	**1.94**	1.16–3.26	0.49	0.07–3.64	**1.96**	1.32–2.91
ECS	1.16	0.89–1.51			1.16	0.89–1.52			1.47	0.93–2.34			1.46	0.97–2.22
Margin <5 mm	**1.76**	1.37–2.27	1.29	0.66–2.53	**1.85**	1.41–2.43	**4.28**	1.18–15.53	1.19	0.68–2.08	1.05	0.36–3.03	**1.85**	1.20–2.86
Late stage	**2.28**	1.71–3.03												

Abbreviations: RMT, retromolar trigone; HR, hazard ratio; CI, confidence interval; WD, well differentiated; MD, moderately differentiated; PD, poorly differentiated; PNI, perineural invasion; LVI, lymphovascular invasion; ECS, extra-capsular spread.

**Figure 3:**
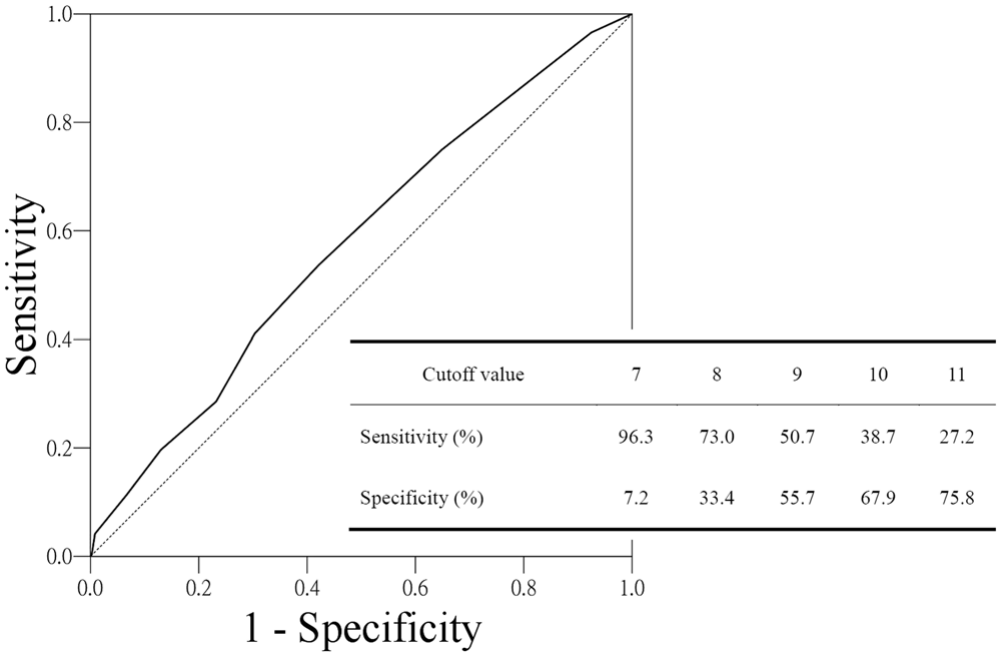
Receiver operator characteristic (ROC) curve for cutoff analysis of score in patients with loco-regional recurrence. The area under the curve is 0.547. The table represents statistical parameters calculated for different cutoff values to predict loco-regional recurrence.

When applying the scoring system on our patients, the data are shown in Table 5. The result showed progressively increasing rate of loco-regional recurrence from lower score to higher score (*P* = 0.003). For the ease of further analyses, we used a receiver operator characteristic (ROC) curve to identify a proper cut-off point at which to divide the patients into two groups. The ROC curve was drawn according to the sensitivity and specificity that the score could discriminate the status of loco-regional recurrence. The longitudinal axial represents “sensitivity” whereas the horizontal axial represents “1 – specificity” (Fig. 3). The area under ROC curve was 0.547 (*P* = 0.004). The sensitivity and specificity of scores were listed and the cut-off point was chosen when the sensitivity and specificity were both as high as possible (Fig. 3). Using a logistic regression model, patients with score >9 had a higher risk of developing local recurrence when compared to patients with score <=9 (odds ratio, 1.294; 95% CI, 1.037–1.614; *P* = 0.022). In addition, patients with score > 9 had a poor prognosis when compared with that of patients with score <=9 (5-year DSS, 51.89% vs. 85.47%; Log-rank value, 172.42; *P* < 0.001).

**Table 5:** Results of application of the scoring system to 1383 patients.

Score	No. of patients (% in column)	No. of loco-regional recurrence (%)
7	82 (5.9)	18 (22.0)
8	349 (25.2)	114 (32.7)
9	308 (22.3)	109 (35.4)
10	168 (12.1)	59 (35.1)
11	127 (9.2)	56 (44.1)
12	134 (9.7)	44 (32.8)
13	98 (7.1)	41 (41.8)
14	88 (6.4)	32 (36.4)
>15	29 (1.8)	16 (55.2)

## Discussion

Most previous studies on the prognostic factors of OCSCC do not analyze subsites separately^5, 6, 8–12, 15^. Although no significant differences existed among various subsites in the 5-year DSS, here we found the prognostic impact of pathological features was subsite-dependents. A previous study has reported significant differences in the failure pattern between tongue and buccal carcinomas^13^. Lin *et al*. in their study on advanced-stage oral cavity cancer patients receiving definitive radiotherapy also found that the primary tumor site was a prognostic factor^16^. Different prognostic factors of subsites may be related to the diverse molecular genetic pathways. Mahdey *et al*. compared tongue and buccal SCC and found a higher level of positive amplification in cyclin D1 in tongue SCC (88%) as opposed to that in buccal SCC (56%)^17^. Besides, the genetic expression profiling was reported to be different by the anatomic site of the primary tumor^18, 19^. Another possible explanation is that the compact and complex anatomy of the oral cavity leading to different tissue composition among various subsites. In addition, different subsites might also have dissimilar vulnerability of tumor invasion^20^.

In a population-based cohort study on early stage oral SCC, patients have a higher death rate when presented with poorly differentiated or undifferentiated tumors in contrast to those with well differentiated tumors (HR, 2.7; 95% CI, 1.72–4.11)^21^. Histological grade was also reported to be associated with positive surgical margins, regional lymph node metastasis, and loco-regional recurrence. In addition, oral SCC with higher histological grades is thought to be more aggressive and likely to become more locally advanced at the time of diagnosis^21^. However, most investigators do not consider histological grade as a good indicator for the outcome or treatment response^22^. The discrepancy in views is probably related to the distribution pattern of SCC at various subsites. In Taiwan, the distribution pattern is different from that in the Western countries: as the majority of OCSCC are tongue and buccal SCC which are most likely caused by the prevalent betel quid chewing in the population^13^. We have found that the histological grade was independently associated with survival of OCSCC patients. However, when all patients were analyzed, the prognostic impact of histological grade on 5-year DSS was only significant at the subsite of buccal mucosa.

The mechanism of PNI is still unclear. It remains debatable regarding the beneficial effect of adjuvant radiotherapy in patients of early head and neck cancers presented with PNI^23^. One cause of controversy is the subjectiveness in the interpretation of PNI^8^. The discrepancy in results across studies is inevitably due to differences in study design, study population, methodology such as in the evaluation of PNI and the extent of nerves involved^8, 15^. Although tumor thickness, which is one of the main prognosticators, was not included in final analysis in the current study, a previous study found that PNI correlated well with the tumor thickness^24^. Another reason why PNI was an independent poor prognostic factor of early stage OCSCC in the current study might be due to different treatment modalities. Barry *et al*. in their case-matched study concerning oral squamous cell carcinoma patients with intermediate risk of recurrence found that postoperative radiotherapy significantly improved loco-regional control rate^25^. PNI was reported to be strongly correlated with aggressive tumor behavior, disease recurrence, and decreased survival. Various molecular markers have been associated with PNI, including apoptosis-associated Speck-like protein containing a CARD (ASC), HMGA2 and MIF^26, 27^. Nevertheless, further research is warranted before targeting PNI as a part of advanced cancer therapy.

LVI implies that a large number of tumor cells have entered the lympho-vascular compartment and consequently leads to a higher chance of regional and distant metastasis^22^. Though the importance of LVI in the prognosis of other solid tumors is well documented, little attention has been paid on the relationship between LVI and survival rate of OCSCC patients^10^. A previous study of the oral cavity reported that tumor emboli are less likely to form in the small-caliber lymphatics of superficial tissues than in the larger caliber lymphatics of deep tissue^22^. This can explain for the poor prognostic value of LVI in late stage OCSCC found in our study.

It is interesting to note that we found a prognostic value of ECS with OCSCC only in univariate analysis. The survival impact of ECS was weak in multivariate analysis. The prevalence of ECS is known to be related to the size of primary tumor^11^. Patients with ECS were reported to have a better survival following treatment with postoperative chemo-radiation as compared to treatment with only postoperative radiation^28^. Taken together, aforementioned findings might be the reasons why ECS is not associated with poorer survival in OCSCC patients in the current study.

Although inadequate surgical margin is associated with a poorer 5-year DSS, the survival disadvantage was only significant in some portion of our patients with OCSCC. When we divided the patients into various groups according to their primary subsites, surgical margin less than 5 mm was a poor prognosticator for patients with gum, floor of mouth, tongue, and buccal SCC. Inadequate margin in patients with oral SCC was reported to have a negative impact on survival^22^. In contrast, Hasegawa *et al*. reported a contradicting finding that the status of surgical margin is unrelated to survival^6^. None of the above studies showed that inadequate margin would increase the possibility of loco-regional recurrence. The discrepancy in results could be related to the different distribution patterns of subsites within these studies. In addition, recent advances in multimodalities treatment protocol might also suppress the effect of inadequate margin. Although the assessment of the status of the surgical margins is subject to numerous errors of interpretation, it should be emphasized that adequate margins during surgical excision of primary tumor is still crucial in spite of the findings in the current study.

Development of outcome prediction models in cancer is not new in the literature. Arora *et al*. in their study on early-stage OCSCC patients established the Aditi-Nuzhat Lymph-node Prediction Score (ANLPS) System to predict neck lymph node(s) metastasis by using histopathologic parameters including depth of invasion, pattern of invasion, PNI, histological grade, LVI, lymphoid response, and tumor budding^29^. Other grading systems, such as Brandwein-Gensler, Bryne, Anneroth, and Martinez-Gimeno score system, have been proposed for evaluation of prognosis or neck metastasis in OCSCC patients^29–31^. These models can provide supplementary predictive information aside from the TNM staging system for OCSCC patients. Nevertheless, a major drawback with classifications based on histologic features is frequent lack of inter-observer agreement which limits the clinical application of subjective histological categorizations. Furthermore, many versions of modifications in the literature may imply the dilemma of inconsistency of these complicated malignancy grading systems in their predictive value^31^.

We must also point out limitations of our study. First, we adopted a retrospective design which is not bias-free. Second, the external validity of our findings is limited by the sample collected exclusively within a single institute. Third, due to incomplete records, tumor thickness or depth was not analyzed. Lastly, while the therapeutic guidelines are standardized in our institute, individual differences among patients were uncontrolled.

In conclusion, the prognostic impact of pathological features from various subsites on the survival of OCSCC patients is not the same. First, PNI was associated with a poor prognosis with early stage oral cavity SCC especially in patients with primary at the tongue. In addition, LVI was related to a poor survival rate with late stage OCSCC especially in patients with primary at the buccal mucosa. Further investigation is warranted to validate our finding in a multicenter study. Grouping the different markers to establish a prognostic scoring system may provide more accurate evaluation of the prognosis in OCSCC patients.

## Materials and Methods

This retrospective study was approved by the Institutional Review Board (IRB) of Taichung Veterans General Hospital (TCVGH) (date: August 17, 2015, approval number: CE15226A). Due to the treatment has been completed and the data were collected retrospectively, informed consent was exempted by the IRB of TCVGH. We reviewed over 2,000 medical charts of patients admitted for the treatment of oral cavity cancer in the TCVGH from January 2004 to December 2014 with the observation ending on December 31, 2015. Therapeutic protocols for all patients were in accord with the consensus guideline of the oral cancer team of TCVGH. We chose patients received surgical excision of primary tumor plus neck dissection as their first treatment only. Adjuvant treatment, such as radiation along or concurrent chemo-radiotherapy, was arranged in patients with risk factors, which was basically in accordance to the guidelines of National Comprehensive Cancer Network. Data were validated by cross-linking with the Cancer Registry Dataset from the TCVGH.

The variables for analysis were age, gender, personal habits, tumor stage, treatment modalities, tumor location, histological features (tumor grading, PNI, LVI, ECS), and margin status. The histological differentiation followed the WHO grading system and the pathological staging followed the 2009 criteria of the American Joint Committee on Cancer. PNI was described in accordance with the College of American Pathologists (CAP) protocol without quantification whereas LVI was categorized based on the presence of tumor nests within or adjacent to the endothelial cell lining of the lymphovascular space as well as tumor cell permeation in the endothelium-lined vessels^9^. ECS was defined as tumor infiltrating beyond the lymph node capsule^11^. The definition of local recurrence is the pathological evidence of SCC that was found close to the location of index tumor after comprehensive treatment. Distant metastasis was assessed mainly by image study. Survival duration was defined as the period from the date of surgery to the date of death or the last date of follow-up in the study.

All relevant histopathological variables were taken together and given a numerical value to build a scoring system. One point represented the lowest risk of mortality, and the score was added with an additional 1 point to denote a higher risk of loco-regional recurrence^29^. The variables include histological differentiation (WD: 1, MD: 2, PD: 3), pathological tumor stage (T1–2: 1, T3–4: 2), pathological nodal stage (N0: 1, N1: 2, N2-N3: 3), PNI (no: 1, yes: 2), LVI (no: 1, yes: 2), ECS (no: 1, yes: 2), and surgical margin (>=5 mm: 1, <5 mm: 2). Eventually, the sum of abovementioned score in each patient ranges from 7 to 16.

### Statistical Analysis

Demographic data were presented as descriptive statistics. The differences of continuous variables among subgroups were compared using analysis of variance, and nominal or ordinal variables were analyzed using the Chi-square test. Survival analysis was done using the Kaplan-Meier method and the differences among subgroups were assessed using the log-rank test. Furthermore, factors that could influence the survival period were examined by the Cox proportional hazard model. All analyses were computed by SPSS for Windows, version 12.1 (SPSS, Chicago, IL). Statistical significant level was set at *P* < 0.05.

### Data Availability

All data generated or analyzed during this study are included in this published article.
